# Usability Testing and Adaptation of the Pediatric Cardiovascular Risk Reduction Clinical Decision Support Tool

**DOI:** 10.2196/humanfactors.5440

**Published:** 2016-06-21

**Authors:** Pamela A Williams, Robert D Furberg, Jacqueline E Bagwell, Kenneth A LaBresh

**Affiliations:** ^1^ RTI International Social Policy, Health and Economics Research Unit Research Triangle Park, NC United States

**Keywords:** adaptation, cardiovascular diseases, clinical decision support, decision aids, guidelines, mHealth, pediatrics, risk factors, usability

## Abstract

**Background:**

Cardiovascular disease (CVD) is 1 of the leading causes of death, years of life lost, and disability-adjusted years of life lost worldwide. CVD prevention for children and teens is needed, as CVD risk factors and behaviors beginning in youth contribute to CVD development. In 2012, the National Heart, Lung, and Blood Institute released their “Integrated Guidelines for Cardiovascular Health and Risk Reduction in Children and Adolescents” for clinicians, describing CVD risk factors they should address with patients at primary care preventative visits. However, uptake of new guidelines is slow. Clinical decision support (CDS) tools can improve guideline uptake. In this paper, we describe our process of testing and adapting a CDS tool to help clinicians evaluate patient risk, recommend behaviors to prevent development of risk, and complete complex calculations to determine appropriate interventions as recommended by the guidelines, using a user-centered design approach.

**Objective:**

The objective of the study was to assess the usability of a pediatric CVD risk factor tool by clinicians.

**Methods:**

The tool was tested using one-on-one in-person testing and a “think aloud” approach with 5 clinicians and by using the tool in clinical practice along with formal usability metrics with 14 pediatricians. Thematic analysis of the data from the in-person testing and clinical practice testing identified suggestions for change in 3 major areas: user experience, content refinement, and technical deployment. Descriptive statistical techniques were employed to summarize users’ overall experience with the tool.

**Results:**

Data from testers showed that general reactions toward the CDS tool were positive. Clinical practice testers suggested revisions to make the application more user-friendly, especially for clinicians using the application on the iPhone, and called for refining recommendations to be more succinct and better tailored to the patient. Tester feedback was incorporated into the design when feasible, including streamlining data entry during clinical visits, reducing the volume of results displayed, and highlighting critical results.

**Conclusions:**

This study found support for the usability of our pediatric CVD risk factor tool. Insights shared about this tool may be applicable for designing other mHealth applications and CDS tools. The usability of decision support tools in clinical practice depends critically on receiving (ie, through an accessible device) and adapting the tool to meet the needs of clinicians in the practice setting.

## Introduction

By 2020, cardiovascular disease (CVD) is projected to rank first in frequency among causes of death, years of life lost, and disability-adjusted years of life lost worldwide [1]. Because risk factors and behaviors that begin in youth can contribute to CVD later in life, prevention needs to start with children and teenagers [2]. Recognizing this need, in 2012 the National Heart, Lung, and Blood Institute (NHLBI) [3] released its 402-page, evidence-based “Integrated Guidelines for Cardiovascular Health and Risk Reduction in Children and Adolescents.” The comprehensive guidelines describe CVD risk factors that clinicians should address with patients from birth through 21 years of age, and with their families. The guidelines also include recommendations for clinicians on CVD risk factors, such as diet, physical activity, tobacco, blood pressure (BP), lipids and lipoproteins, and overweight and obesity, as well as the influence of family history on risk factor management. However, barriers related to lack of knowledge, effective systems, and support often delay uptake of new guidelines by clinicians [4]. Additionally, clinicians frequently have multiple prevention topics to discuss with patients [5], which may leave little time to add exploring CVD risk factors during primary care visits [6].

To overcome some of the barriers and to support clinicians in implementing the NHLBI CVD guidelines, we developed a comprehensive, multifaceted intervention that includes practice-based clinical champions, monthly collaborative webinars to support practice change, and a tool kit to support guideline implementation. The tool kit comprises a patient and family workbook to support patients in making behavior changes, guideline summary materials that include recommendations for clinicians, and a clinical decision support (CDS) tool [7-10]. CDS tools can improve clinician adherence to guidelines [11]. To assist clinicians in prioritizing the topics most important to individual patients to reduce their specific CVD risk, we developed the Pediatric Cardiovascular Risk Reduction CDS Tool, which provides a concise presentation of guideline information and tools to help clinicians perform the complex assessment of CVD risk factors within the clinical workflow. The tool allows clinicians to input patient and family history, determine guideline-specific recommendations for individual patients, and calculate both individual and combined body mass index (BMI) and BP percentiles. It also supports interpretation of specific laboratory results, including lipid screening values, for planning targeted follow-up visits [10].

The overarching aim of the CDS tool is to provide clinicians at the point of care with actionable, individually relevant recommendations for patients drawn from the comprehensive NHLBI CVD guidelines. We utilized a user-centered design approach in which users participated in pretesting and were involved with refinement of the design throughout the entire development process [12], including creating the content [8], designing and programming [7], pretesting (discussed in this paper), and conducting the experimental study [13]. Overall, we used a feature-driven development approach where primary components of the CDS tool were initially developed and tested independently, refined [7,8,14] and progressively integrated, and tested again.

After we created the content for the CDS tool, we developed other elements using key user-interface and user-experience design principles such as giving the user control, empowering the user, and allowing exploration and browsing; providing immediate feedback and the option of help at any point, and defining terminology used in the app; keeping the interface consistent, with active buttons in the same place throughout [12]; and incorporating actionable feedback related to the user experience and content refinement. We discuss these elements in this paper as we describe the process we used to pretest and adapt the CDS tool to help clinicians implement the guidelines. Research has shown the utility of conducting usability testing to adapt tools to maximize usability [15]. The aim of this study was to examine the usability of the Pediatric Cardiovascular Risk Reduction CDS Tool.

## Methods

**Figure 1:**
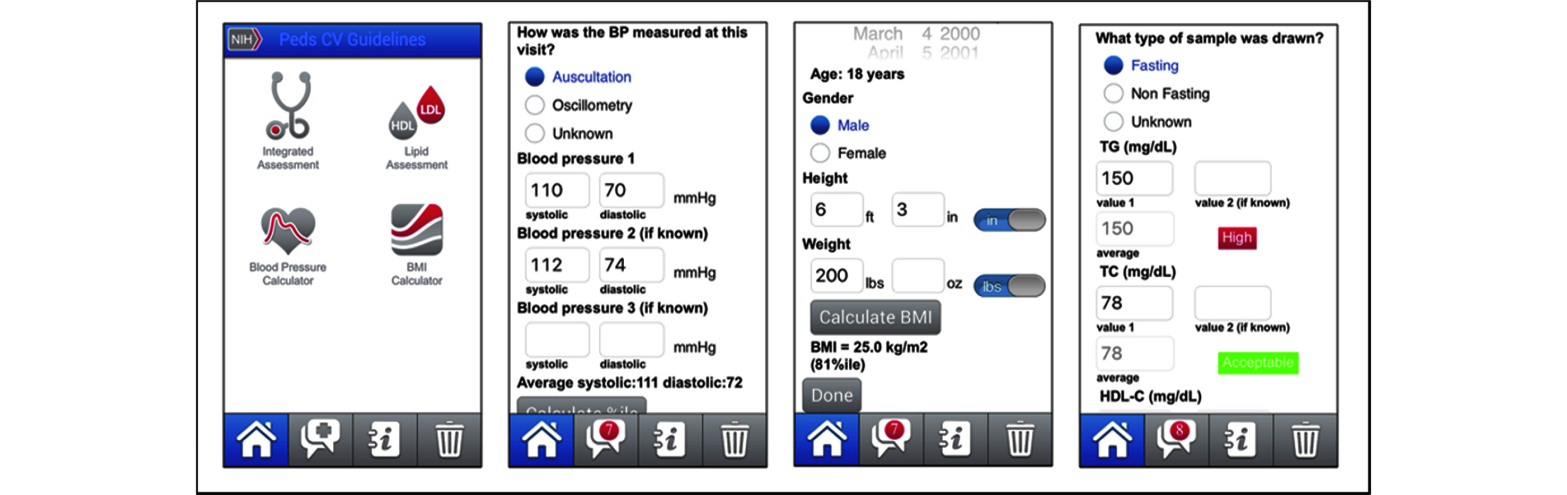
Screenshots illustrating (from left to right) (1) the 4 modules available at login: integrated risk assessment, body mass index (BMI) and blood pressure (BP) calculators, and lipid management instrument; (2) the BP calculator input screen; (3) the BMI calculator input screen; and (4) the lipid management input screen. HDL-C: high-density lipoprotein cholesterol; TC: total cholesterol; TG: triglycerides.

The Pediatric Cardiovascular Risk Reduction CDS Tool consists of a screening (integrated risk) assessment, BMI and BP calculators, and a lipid assessment instrument (Figure 1) and provides clinicians with a patient summary and NHLBI recommendations based on the patient’s risk factor information input by the user. The integrated risk assessment module asks users to enter data to assess BMI (ie, patient’s date of birth, gender, height, and weight), BP (ie, the patient’s systolic and diastolic BP), and other risk factors (eg, whether the patient has dyslipidemia), and then provides users with a patient summary and NHLBI recommendations related to family history, nutrition and diet, physical activity, tobacco exposure, lipids, and overweight and obesity. It also provides supportive actions to take, based on the patient’s risk factor information.

For the BMI and BP calculators and the lipid assessment instrument, if the user has already entered information in other modules (eg, integrated risk assessment), then the app will display the relevant, previously entered data in this module (eg, for the lipid assessment and BMI calculator, the patient’s date of birth, gender, height, and weight). In instances where the user is only interested in using the BMI calculator, BP calculator, or lipid assessment, the user is asked to enter this information. For the lipid assessment, the user is asked to input the type of sample that was drawn (with response options of fasting, nonfasting, and unknown) in addition to BMI. For each of the modules, the user is automatically moved to the recommendations screen showing a patient summary of the data entered and the recommendations, after indicating that the user has finished entering the data.

The tool is a native Android and iOS app that clinicians can use on smartphones or tablet devices to evaluate patient risk, recommend healthy behaviors to prevent the development of risk, and carry out complex calculations to determine the appropriate interventions, as recommended by the NHLBI guidelines. The development of the app content [7,8], implementation protocol [10], and results of an 18-month, cluster randomized trial in 32 clinical practices are described elsewhere [13].

### Approach

We used an iterative process of designing, testing, and revising throughout the design and development life cycle for the screening instrument, the validated BMI and BP calculators, and the lipid assessment instrument [7]. Pretesting, conducted after initial development of the app, was completed in 2 phases. First, we conducted one-on-one in-person testing with clinicians. Second, at a subsequent phase of testing, we examined the use of the app in clinical practice. Using a quantifiable instrument, we also asked the clinical practice testers about their overall experience with the CDS tool. Both testing cycles were reviewed by RTI International’s Institutional Review Board and deemed exempt because no personally identifiable information was obtained from participants and the data gathered were used for systems research.

### In-Person Testing

**Figure 2:**
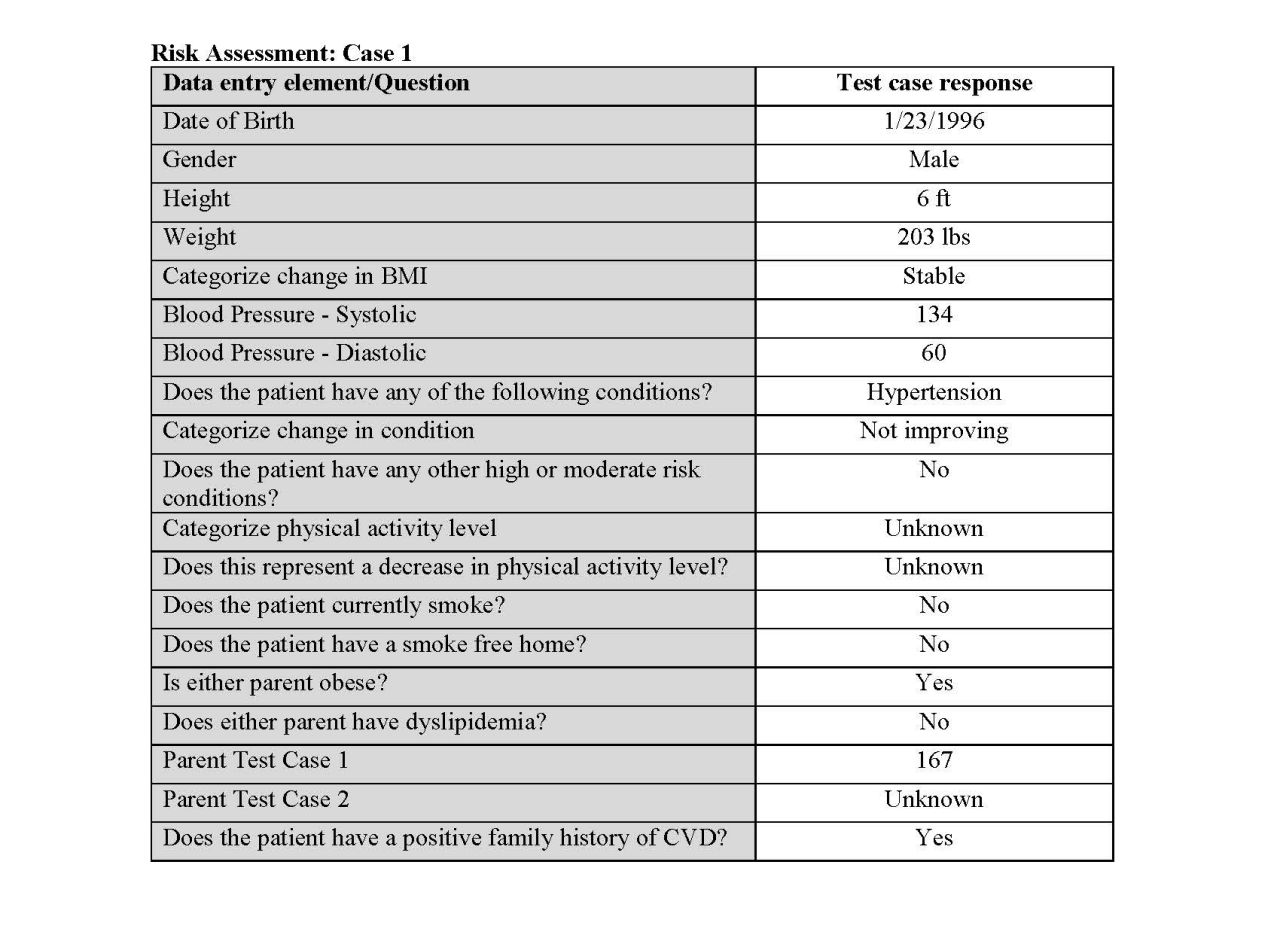
Example of a test case. BMI: body mass index; CVD: cardiovascular disease.

Based on current recommendations from evidence-based user-experience research [16,17], we initiated in-person usability testing by recruiting a convenience sample of 5 clinicians from 2 local universities in the Raleigh-Durham, North Carolina area, using a snowball recruiting approach. A member of the research team conducted one-on-one testing, which lasted for approximately 1 hour. Each participant was given a brief overview of the app and 4 test cases to complete—one each for the screening instrument and the 3 validated calculator modules that the app supports. Test cases were authored by 1 of the authors and CDS product owner (RDF) with domain expert oversight by another of the authors (KAL), which were then reviewed and approved by the chair of the NHLBI Expert Panel. Together, the test cases (Figure 2) represent the clinical scenarios and essential frequent tasks for which the tool would be used. Participants were instructed to enter the data as shown in the scripted scenario using a test device without assistance and to “think aloud” as they went, as is typical in usability testing [18]. The testing sessions were audio-recorded, capturing participants’ verbal feedback. We did not offer participants financial incentives for their participation.

### Testing in Clinical Practice

Because the CDS tool is designed primarily for use by pediatricians, we recruited participants for in-clinic usability testing through the American Academy of Pediatrics (AAP) Quality Improvement Innovation Network member listserv. Members of this professional group are particularly relevant to sample because participants are board-certified pediatricians who are specifically interested in testing practical tools that improve the quality of care for children and their families. A total of 29 clinicians responded to the recruitment email—which invited clinicians to attend a half-hour webinar detailing the project and CDS app—and agreed to use the app in at least 5 patient-encounter scenarios during a 2-week period. We did not offer any financial incentives to clinicians for their participation. We made the tool available to all interested participants through TestFlight, a Web service through which the research team managed access to prerelease versions of the app. Among the 29 initial clinician responders, 19 clinicians downloaded the app and 14 clinicians provided feedback on their user experiences over the 2-week testing period. We gave participants the opportunity to provide immediate, asynchronous feedback on their user experience in the form of unstructured comments via email, telephone, or short message service during the testing period rather than waiting until the end. We included the telephone number and email address of a research team member in the “About” section of the app so that participants could easily provide such feedback. On completion of testing, we instructed participants to delete the app.

A total of 14 participants completed the 10-item System Usability Scale (SUS) [19] questionnaire, which was administered electronically (Textbox 1), to capture their overall experience with the app. To calculate the SUS composite score of the overall usability, we summed the score contributions from each item, which ranged from 1 to 5. For items 1, 3, 5, 7, and 9, the score contribution is the scale position (eg, 4=agree) minus 1. For items 2, 4, 6, 8, and 10, the contribution is 5 minus the scale position (to account for the negative phrasing of these questions). We then multiplied the sum of the scores by 2.5 to obtain the overall SUS composite score, with a possible range from 0 to 100 [19].

**Items in the System Usability Scale (response options ranged from 1=strongly disagree to 5=strongly agree).**

1. I think that I would like to use this clinical decision support (CDS) app frequently.

2. I found the CDS app unnecessarily complex.

3. I thought the CDS app was easy to use.

4. I think that I would need the support of a technical person to be able to use this CDS app.

5. I found the various functions in this CDS app were well integrated.

6. I thought there was too much inconsistency in this CDS app.

7. I would imagine that most people would learn to use this CDS app very quickly.

8. I found the CDS app very cumbersome to use.

9. I felt very confident using the CDS app.

10. I needed to learn a lot of things before I could get going with this app.

In addition to the composite score, we assessed the learnability and usability subscales by analyzing the average responses to learnability (questions 4 and 10 in Textbox 1) and usability items (the remaining 8 questions in Textbox 1) [20]. The learnability and usability subscale scores each have a possible range from 1 to 5.

We also asked users to respond to a few additional questions, including (1) an open-ended question asking “How many times did you use the application during a patient encounter?”; (2) “In what type of patient visit did you use the application?”—with response options of well-child visit, sports physical, weight or obesity follow-up, BP follow-up, lipid follow-up, and other (please specify); and (3) “Which component of the application did you use?”—with response options of integrated assessment, lipid assessment, BP calculator, BMI calculator, and none.

### Data Analysis

We examined the feedback from in-person and clinical practice testers to identify patterns and relevant themes, with the aim of gathering actionable suggestions to revise the CDS tool. We entered the qualitative data into a matrix that segmented clinicians’ comments by comment type, such as positive comment or suggestion for improvement. This common technique in qualitative research enables researchers to organize data to identify commonalities and variations that emerge in comments [21,22]. In this study, 2 of the authors (PAW and RDF) independently reviewed comments to identify relevant patterns and themes, using a coding scheme developed by 1 of the authors (PAW). We coded comments from the in-person and clinical practice testers as being a positive comment, a negative comment, or a suggestion for improvement or change. The same author (PAW) reviewed any discrepancies in interpretation and together 2 of the authors (PAW and RDF) made a final determination.

## Results

### In-Person Testing

A total of 5 clinicians (4 outpatient and 1 inpatient) participated in testing the CDS tool. Each clinician had been actively practicing for more than 2 years. All participants practiced at large academic medical centers. The testers were either general pediatricians, internal medicine physicians who saw a large number of pediatric patients, or pediatricians with subspecialties in nephrology or endocrinology.

Most of the testers’ comments conveyed during the think-aloud sessions involved suggestions for improvements or changes; a few comments were simply compliments, with 9% (5/58) positive comments; and there were no (0%) negative comments. Thematic analysis of the comments from the in-person testers showed that suggestions for improvements or changes fell into 3 categories: (1) layout, navigation, and/or the user experience (41%, 24/58); (2) content refinement (41%, 24/58); or (3) technical deployment (9%, 5/58).

Suggestions for changes to the app related to the user experience and reflected individual preferences for the default display and functionality, such as:

Does not like that the alphabet is the default keyboard, would prefer number pad.

Default to open for the recommendations and supportive action.

Content-related suggestions for changes to the app pointed out areas that needed clarification, particularly related to the information conveyed in the recommendations regarding Estimated Energy Requirement (EER) presented in the patient summary and NHLBI recommendations in the obesity risk section.

Not sure what the EER is. Would like to calculate that in the app.

EER, a pediatrician may not know what this means.

For children with out-of-range BMIs, we provided EER language as part of the recommendations. However, whereas nutritionists understand EER, clinicians typically do not. Consequently, we provided support terminology as a design enhancement. Nonactionable user input included specific criticism of the guideline content, which we were not at liberty to revise; for example:

Shorten the Overweight/Obesity Recommendations.

Consolidate the Tobacco Exposure recommendation with Family History Recommendations.

The few deployment suggestions provided meaningful ideas to improve the usability of the CDS tool for clinicians:

Would like to be able to print physical activity and nutrition/diet recommendations for the patient to take home.

Would like to email the patient [the] patient summary, activity, diet and personal risk factor information.

However, some user input was not actionable because it conflicted with the intended design for a freestanding application; for example:

This information is redundant to the information available in/entered in the EHR [electronic health record].

When considering the screening instrument and the 3 validated calculator modules as well as the functions of the tool, the majority of suggestions for improvement (59%, 34/58) were in response to the content and display recommendations provided by the app based on the patient’s risk factor information input by the user. Common themes included the length of text displayed and the formatting of text, such as:

Supportive action in Family History should only be displayed one time.

Change blood pressure to number spinner rather than number data entry.

Additionally, 28% (16/58) of comments were suggestions for improvement that could be applied to all modules in the app and spoke to the importance of keeping the interface consistent:

When in landscape, the font size changes.

Would like to see metric and standard units displayed on the same screen without having to toggle between the two.

A small number of comments related to only 1 specific module: only 9% (5/58) of the comments related to the integrated assessment and only 1 comment each (<1%) related to the BMI, BP, and lipid modules.

### Testing in Clinical Practice

All 14 in-clinic testers were actively practicing pediatricians whose patient population was more than 80% pediatric. Although 2 participants elected to provide limited, asynchronous feedback via email and telephone during the testing period, most of the input was submitted by all clinicians after completion of the 2-week testing period. The app was used between 1 and 20 times per participant during the testing period, with an average of 7 uses. Participants reported using the app most frequently in well-child visits (87%, 13/15), followed by weight or obesity follow-ups (53%, 8/15), BP follow-ups (40%, 6/15), sports physicals (40%, 6/15), lipid follow-ups (20%, 3/15), and other types of visits (20%, 3/15). The most commonly used modules in the app were the integrated assessment (86%, 13/15) and BP calculator (86%, 13/15), followed by the BMI calculator (73%, 11/15), lipid assessment (47%, 7/15), and none (0%). The results of the SUS data analysis showed that general reactions toward the CDS tool were positive, given the average score of 81, meaning the app was viewed as “above average” with respect to usability (as defined by a score >68; [20]). The learnability subscale average of 1.53 showed that most participants did not think they would need to learn more or require technical support to use the app. Additionally, the 8-item usability subscale average of 3.36 showed that most participants rated the usability of the CDS tool favorably [20].

Consistent with the results from the SUS, general reactions toward the CDS tool were positive, with 35% (6/17) positive comments and no (0%) negative comments; for instance:

Great app - would use it at most visits.

Users particularly appreciated the app’s feature of calculating the BP percentile based on the NHLBI’s BP tables for children and adolescents [23]:

Overall very easy to use and helpful to not have to look up BP values on the chart.

Thematic analysis of the comments from the clinical practice testers showed that suggestions for improvements or changes fell into 2 categories: (1) layout, navigation, and/or the user experience (24%, 4/17), or (2) content (41%, 7/17). Suggestions for changes to the app related to the user experience and to changing the app to give the user control and empower the user, especially clinicians using the app on the iPhone:

When entering numbers on the iPhone, I had to click past the alpha keyboard to get to the numbers. Other apps I have used have the number keypad come up first!

The touch screen did not respond easily to touch and many features were very erratic in their scrolling, such as dates.

The associated algorithms are great but difficult to properly visualize on the small screens of the smartphones.

Content-related suggestions for changes to the app focused on the amount of information conveyed in the recommendations. Sometimes users thought the app provided too much information and they suggested reorganizing the information to make it more succinct:

The recommendations should be narrowed down using the answers entered. Otherwise there are too many and it becomes cumbersome to use.

The recommendations were too lengthy to be useful in a clinical visit.

The recommendation sat the end of the assessment are very wordy. It is a lot of information and the recommendations are important, so streamlining that will mean more people use the app.

Nonactionable user input included specific criticism of the guideline content, which we were not at liberty to revise, for example:

My only problem with the app was it gave too many repetitive recommendations for healthy children with no risk factors. For example, I would enter data for a child with 25% BMI, enter nonsmoking for child and parents, normal values for parent cholesterol values and normal answers to questions about family risk factors, and it would still recommend I ask about smoking and family risk factors after that.

In other instances, testers wanted additional information and information better tailored to the individual patient:

I really wanted to know how to intervene when I had abnormal lipids, but that wasn't easily accessible.

During the integrated assessment it seemed to give the general guidelines more than telling what to do with this specific patient.

## Discussion

### Principal Findings

Clinical decision support tools can improve clinician adherence to guidelines [11,13]. This paper demonstrates the process and value of testing and adapting a CDS tool to assist clinicians in implementing the NHLBI “Integrated Guidelines for Cardiovascular Health and Risk Reduction in Children and Adolescents” [3], using a user-centered design approach. Testers generally responded positively toward the CDS tool. They particularly appreciated the app’s feature of calculating the BP percentile based on the NHLBI’s BP tables for children and adolescents. Testers suggested changes to the app related to user experience, content refinement, and technical deployment. The majority of the suggested changes centered on the content and display of the recommendations for clinicians, including making the app more user-friendly for clinicians using the app on the iPhone and reorganizing and tailoring the recommendations. These findings are similar to those from other usability studies of decision support tools, which often show that testers recommend clearer, more concise content; a more user-friendly layout design; and improvements in navigation [15,24,25] to enhance tools.

However, we employed a user-centered approach and systematic process to develop the Pediatric Cardiovascular Risk Reduction CDS Tool that many developers do not implement. Our approach illustrates a field-proven method for soliciting expert user input from a geographically distributed sample of difficult-to-reach participants. Collaboration with the AAP, the credentialing body for all board-certified pediatricians in the United States, enabled access to members of the AAP’s Quality Improvement Innovation Network. This network was established to provide a standard mechanism for developing practical and usable measures, tools, and strategies for the practicing pediatrician in a primary care practice as well as the pediatric hospitalist in the inpatient setting. Engagement with this professional association provided a point of entry to a practical working laboratory for gathering pediatrician input based on their use of the CDS in real-world patient encounters.

We incorporated tester feedback into the Pediatric Cardiovascular Risk Reduction CDS Tool design when feasible and applicable. Actionable user input focused mainly on matters of user experience and recommendations to streamline the use of the app during clinical visits. We received feedback that translated into changes in data entry, presentation of recommendations, and presentation of critical results. For example, testers recommended simplifying data entry, which resulted in our asking fewer questions and implementing persistence of forms-based variables across all of the instruments, meaning data entered in 1 tool would carry over to another.

Testers also recommended reducing the volume of results displayed, which informed the restructuring of the recommendations layout using design patterns. Consequently, we used faceted navigation and presentation of information to provide an integrated, incremental search-and-browse experience to increase tailoring and refinement of the results presented for an individual patient. The more information—such as social and family history, known risk factors, and clinical observations—that the clinician enters in the CDS tool, the more facets are used progressively to refine results, eliminating extraneous information by narrowing search results. This empowers the user by not forcing clinicians to enter data for all of the variables on a screener page. Consistent with user input regarding the burden of data entry, we did not force data entry and enabled clinicians to figure out how much data they wanted to enter in the screener.

User input on the presentation of recommendations also led to refinements to improve readability of the content on the summary results page. Consequently, we reduced the amount of text and used input from testers to make the recommendation language more actionable (eg, “Measure fasting lipid panel 2x and average results”) rather than providing a more detailed recommendation for follow-up.

Additionally, suggestions led to us providing immediate user feedback with the addition of a color-coding feature (red, yellow, green) to highlight critical or elevated results, such as highlighting when out-of-range or borderline results emerged for BMI, BP percentile, and lipids. Finally, nonactionable user input included specific criticisms of the guideline content, health information technology deployment, and workflow issues, all of which were beyond the scope of this CDS design.

### Limitations

One limitation of the study is that we tested the tool using clinicians who were interested in quality improvement and motivated to adopt decision aids. Consequently, participating clinicians may have been more readily amenable to using the CDS tool or more adept at using it than clinicians without this background, experience, or interest. This, and other individual factors that we did not examine (eg, age), may affect the way clinicians adopt the CDS tool. Certainly testing with more “typical” clinicians who may be less technology savvy would be valuable in future work to identify and address as many usability issues as possible and to ensure that the app is user-friendly for those less familiar with the technology.

Another limitation is that the CDS tool was a part of a multifaceted intervention, which limited our ability to assess individual clinician engagement with the app in more detail. During our testing in clinical practice, we did not measure the duration of time spent with the app during the patient visit. Further, we did not ask clinicians to comment on how the CDS tool affected the patient visit and the patient-provider relationship during the clinical practice testing, which would ultimately be a contributing factor to whether or not the CDS is adopted in the clinical setting.

We also were unable to retest the refined design after incorporating user feedback because of resource constraints. However, the user feedback described in this paper was applied to changes made in the final version of the Pediatric Cardiovascular Risk Reduction CDS Tool and testers were informed of how their input from usability testing had been incorporated in the final build.

Additionally, for the development of this tool, we were responding to a request for a freestanding decision support application. Consequently, we deliberately built it in the context of physician maintenance of certification criteria and to support the trend of individual physicians and staff to use personal mobile phones or other devices. The advantage of this is that the tool is highly portable, which makes it easier to use within the clinical workflow. However, the lack of integration with electronic health records (EHRs) requires additional effort to enter relevant clinical information. This may limit the uptake of this freestanding tool. As EHRs become more prevalent, integration will likely become more of an issue. In the future, developers should consider the relative benefit of building a Web application with an application program interface library, including clear standards for the exchange of clinical variables and bidirectional communication functions between the decision support application and other clinical information systems. Future work should also concentrate on how the CDS will integrate into the broader health care ecosystem.

Finally, while this paper focused on the usability testing, rather than the implementation and effectiveness (see [9,10,13] for details on these aspects) of the CDS tool, it is worth noting that questions remain about the specific components of CDS tools that are effective, the impact of CDS tools on patient outcomes and clinical workload, and clinician preferences for certain CDS features [26-29]. This CDS tool was intended to improve guideline uptake. Whereas prior research has shown that many CDS tools improve clinician adherence to guidelines [11] and other aspects of their performance, the effects of such tools on patient, economic, workload, and efficiency outcomes are understudied [26,27]. However, the Pediatric Cardiovascular Risk Reduction CDS Tool—in combination with the other tools, education, and support that comprised the full comprehensive, multifaceted intervention—was effective in improving adoption of the guidelines [13]. Improved patient outcomes should follow from clinician implementation of these guidelines, if the patient implements the suggested behavior changes. Clinical decision support tools that reduce clinicians’ efforts to digest and impart recommendations have been shown to be central to improving patient care [28], which was a primary benefit of this tool that focused on making a massive set of NHLBI guidelines accessible to clinicians. Other features of successful CDS tools that our CDS tool did not incorporate include providing advice for patients and clinicians at the same time to support improved patient-provider communication or shared decision making and requiring clinicians to give reasons when overriding CDS recommendations [29].

### Conclusions

This study assessed the usability of the Pediatric Cardiovascular Risk Reduction CDS Tool by clinicians. Testing through both one-on-one in-person testing using a "think aloud" approach and in-practice use of the tool along with formal usability metrics revealed ways to optimize the tool related to the user experience, content, and deployment. Although this paper focuses on a CVD tool, the insights that we shared about the reactions to testing and adapting this tool may be applicable to the design of other mobile health apps and CDS tools. Future work should bear in mind the benefits of integration with EHRs as they become more prevalent in the coming years.

## Abbreviations

AAP: American Academy of Pediatrics
BMI: body mass index
BP: blood pressure
CDS: clinical decision support
CVD: cardiovascular disease
EER: Estimated Energy Requirement
EHR: electronic health record
NHLBI: National Heart, Lung, and Blood Institute
SUS: System Usability Scale
